# A longitudinal study of changes in smart phone addiction and depressive symptoms and potential risk factors among Chinese college students

**DOI:** 10.1186/s12888-021-03265-4

**Published:** 2021-05-14

**Authors:** Xue Yang, Huahua Hu, Chengjia Zhao, Huihui Xu, Xiaolian Tu, Guohua Zhang

**Affiliations:** 1 JC School of Public Health and Primary Care, Faculty of Medicine, The Chinese University of Hong Kong, Hong Kong, China; 2 Department of Psychology, School of Mental Health, Wenzhou Medical University, University Town, Chashan, Wenzhou, Zhejiang 325035 P.R. China; 3 Renji College, Wenzhou Medical University, Wenzhou, China; 4 The Affiliated Kangning Hospital, Wenzhou Medical University, Wenzhou, China; 5 School of Mental Health, Wenzhou Medical University, Wenzhou, China

**Keywords:** COVID-19, Smart phone addiction, Depressive symptoms, Young adults, Longitudinal

## Abstract

**Background:**

The current study aims to track the changes in the levels of smart phone addiction (SPA) and depressive symptoms between pre and during COVID-19 and potential risk factors of among Chinese college students in a four-wave longitudinal study.

**Methods:**

The participants were recruited from a Chinese university (*n* = 195; 58.5% females). The first three-wave surveys were conducted before COVID-19 (during December of Year 1, June of Year 1, and December of Year 2 of their college study; Time 1, Time 2, Time 3), while the fourth survey (Time 4; during June of Year 2 of their college study) was conducted in June 2020 during COVID-19. COVID-19-related factors, including quarantine, lockdown, boredom, emotional loneliness, and social loneliness, were investigated.

**Results:**

The results showed a significant increase in the levels of depressive symptoms and prevalence of probable depression during COVID-19 (69.2%) compared to those 18 months, 12 months and 6 months before COVID-19 (41.5, 45.6, 48.2%) but non-significant changes in SPA. Boredom and emotional loneliness were positively associated with both SPA and depressive symptoms during COVID-19. Social loneliness was also positively associated with depressive symptoms during COVID-19. Quarantine and lockdown were not significantly associated with SPA or depressive symptoms.

**Discussion and conclusions:**

The results highlight that the study population may be a high risk group of probable depression. Future studies should continue to track these mental and behavioral status with the progression of the epidemic. The identified emotional factors could be used to reduce depressive symptoms during COVID-19 and prevent the potential risk of SPA.

**Supplementary Information:**

The online version contains supplementary material available at 10.1186/s12888-021-03265-4.

## Introduction

The COVID-19 pandemic and unprecedented control measures, such as lockdown, quarantine, social distancing, and home confinement [1, 2], may have caused marked changes in our mental and behavioral health within a short timeframe. As a large proportion of the global population hunkers down in isolation, negative emotions (e.g., boredom, loneliness, distress) and excessive use of digital platforms may substantially increase. Thus, problems related to mental health (e.g., depression) and digital lifestyles (e.g., smart phone addiction) may have become significant public health concerns.

It is already evident that the direct and indirect psychological effects of the COVID-19 pandemic and control measures are pervasive and may affect mental health now and in the future (e.g., [3–7]). A number of cross-sectional studies have reported high prevalence of probable depression (assessed by non-clinical measures of depression) during COVID-19 [8–10]. For example, compared to pre-COVID studies, significant increase in prevalence of depressive symptoms among general population have been observed in China (26.9% vs 2.1%) [8, 11], Italy (32.4% vs 6%) [9] and Germany (14.3% vs 5.6%) [10]. However, we did not identify any longitudinal study directly monitoring the changes in depressive symptoms in the same sample over time. We only identified one study analyzing data from the U.S. Census Bureau collected before and during the COVID pandemic which found that depression prevalence in the US increased from 6.6% in 2019 to 24.9% in late May 2020 [12].

On the other hand, as individuals may largely depend on digital platforms for social connection, entertainment, and information sharing during COVID-19, their digital lifestyles may have been changed. However, it is worth noting that protracted periods of isolation, technology-based activity, and limited face-to-face interaction may have intensified digital related behavioral problems, such as smart phone addiction (SPA). SPA refers to the phenomenon characterized by withdrawal symptoms, tolerance, dependence, and social problems when individuals overindulge in smart phone use [13]. In addition, SPA has also been termed mobile phone dependence [14] and problematic phone use [15, 16]. Some studies used these terms interchangeably, while some conceptualized them differently. It has been documented that SPA can lead to severe mental health problems, such as anxiety disorders, depression, higher perceived stress and insomnia [17, 18], and may increase the danger of solidifying unhealthy lifestyle patterns and leading to difficulties to re-adaptation when the COVID-19 crisis has passed [19–21]. Although the expert consensus on the need of preventing such technology-related disorders and behavioral addiction due to COVID-19 has been highlighted by a couple of commentaries, empirical research on these problems is lacking [19–21]. A brief report conducted by Sun (2020) found that 46.8% of the subjects reported increased internet dependence during the pandemic in China [21]. We did not identify any studies on SPA in the context of COVID-19. Monitoring the changes in mental and behavioral problems at different COVID-19 epidemic stages will facilitate early detection and early treatment.

Negative emotions, such as boredom and loneliness, due to the COVID-19 isolation may have substantially increased during COVID-19 [22, 23], and may be important psychological factors of depression and SPA. Boredom refers to a state of relatively low arousal and dissatisfaction, which is attributed to an inadequately and mentally stimulating environment [24]. This state is transitory; a person may be in a state of boredom in one instant or situation and not in the next instant or another situation [24]. Loneliness is defined as “an enduring condition of emotional distress that arises when a person feels estranged from, misunderstood, or rejected by others and/or lacks appropriate social partners for desired activities, particularly activities that provide a sense of social integration and opportunities for emotional intimacy.” (p. 1391) [25]. Both boredom and loneliness are well-documented risk factors of depression in non-COVID contexts (e.g., [26, 27]). Also, the three states frequently co-occur, and measures of the three states are substantially correlated [28]. To our knowledge, no study tested the association between boredom or loneliness experienced and depression during COVID-19. In addition, individuals with great loneliness and boredom may excessively use smart phone as a means of relieving these negative emotions and ameliorating social isolation during COVID-19, which may increase the risk of SPA. According to the general strain theory, negative emotions resulted in external and environmental stress can lead to addictive behaviors and behavioral problems especially when individuals fail to find legitimate ways to manage their negative emotions and feel the need to attack or escape from adversity [29]. The theory has been used to explain a broader range of maladaptive outcomes, especially various forms of addiction, such as substance use and internet dependence in non-COVID contexts [30–33]. We did not identify any studies that examined the associations between boredom or loneliness and SPA.

### The present study

The four-wave longitudinal study aimed to monitor the levels of SPA and depressive symptoms before and during COVID-19 in a sample of college students in China. Furthermore, the study investigated the potential risk factors of SPA and depressive symptoms, including quarantine status, lockdown, and emotional distress (i.e., boredom, loneliness) during COVID-19. It is hypothesized that quarantine status, lockdown, boredom, and loneliness would be positively associated with SPA and depressive symptoms.

## Methods

### Participants

The participants were recruited at Wenzhou Medical University in Wenzhou City, Zhejiang Province, China. The inclusion criteria of this study included: 1) being a first-year college student; 2) willing to participate in the baseline and follow-up studies; and 3) using a smart phone on a daily base. The exclusion criteria included: 1) non-Chinese speaker; and 2) having cognitive impairment to understand the survey questions. Data from the participants who completed four-wave surveys were reported in this study (*N* = 195). About three-fifth of the participants (58.5%) were female, 52.3% were from urban areas, 48.2% lived in one-child family, and 58.4% spent more than 4 h on smart phone daily (Table 1).

**Table 1:** Background characteristics of the participants (*N* = 195)

	n	%
Sex		
Female	114	58.5%
Male	81	41.5%
Residence		
Urban	102	52.3%
Rural	93	47.7%
One-child family^a^		
Yes	94	48.2%
No	100	51.3%
Hours of smart phone use per day		
> 0–4	81	41.6%
> 4–8	104	53.3%
> 8	10	5.1%
Major		
Anesthesia	27	13.8%
Forensic Medicine	29	14.9%
Stomatology	54	27.7%
Chinese Medicine	85	43.6%

^a^1(0.5%) missing value

### Data collection

The four-wave survey was conducted during December of Year 1, June of Year 1, December of Year 2, and June of Year 2 of their college study. We refer to these as the Time 1 (T1), Time 2 (T2), Time 3 (T3), and Time 4 (T4) assessments, respectively. The survey at T1, T2, T3 was conducted before COVID-19 outbreak, while T4 was conducted in June 2020 during COVID-19. All the surveys were conducted in classroom settings. A research assistant with 2-year experience of data collection delivered the survey and explained to the participants that participation was voluntary, and refusals would have no negative consequences. Data confidentiality was guaranteed and only the researchers could access the data. Student ID was collected for data matching. Researchers were not able to access students’ names or other identity information.

### Ethics approval and consent to participate

The informed consent was obtained from all the participants. This study was conducted following the Declaration of Helsinki. The ethical approval of this study was obtained from the Ethics Committee of the corresponding author’s university.

### Measures

#### Boredom

Boredom during COVID-19 was measured by three items of the Multidimensional State Boredom Scale (MSBS) at T4 [34]. The three items were adapted into the context of COVID-19 (i.e., “I feel bored during COVID-19”, “I am easily distracted during COVID-19”, and “Time is passing by slower than usual during COVID-19”). The items are rated on a Likert scale (1 = *strongly disagree* to 5 = *strongly agree*). A higher score indicates a higher level of boredom during COVID-19. The Chinese version has been used in previous studies [35]. The scale reliability was acceptable (Cronbach’s alpha = .70).

#### Loneliness

Loneliness was assessed by the Chinese version of the 6-item De Jong Gierveld Loneliness Scale at T4 [36]. It includes two subscales of emotional loneliness (i.e., “I miss having people around”) and social loneliness (i.e., “There are enough people I fell close to”) [36]. Response options include “no”, “more or less” and “yes”. Higher scores suggest higher levels of emotional loneliness and social loneliness during COVID-19. The Cronbach’s alpha was .69 for the emotional loneliness subscale and .90 for the social loneliness subscale.

#### Smart phone addiction

Smart phone addiction was assessed by the 17-item Mobile Phone Addiction Index Scale at T1-T4 [37, 38]. Sample items of the scale include “You have tried to hide from others how much time you spend on your mobile phone” and “You find yourself occupied on your mobile phone when you should be doing other things, and it causes a problem”. Each item is scored from 1 (*never*) to 5 (*always*), with a higher score indicating a higher level of SPA. The scale showed good reliability and validity in Chinese populations [39, 40]. The Cronbach’s alpha for the scale was .85 at T1, .88 at T2, .91 at T3, and .76 at T4.

#### Depressive symptoms

Depressive symptoms were assessed by the Chinese version of the 20-item Center for Epidemiologic Studies Depression Scale (CES-D) at T1-T4 [41]. The scale was “designed to measure the current level of depressive symptomatology, with emphasis on the affective component, depressed mood” ([42], p. 385). Participants rated how often they experienced the symptoms such as restless sleep and feeling lonely in the past 7 days on a 4-point scale, ranging from 0 (*rarely or none of the time*) to 3 (*almost or all of the time*). The total score ranged from 0 to 60, with higher scores indicating greater depressive symptoms. As typically recommended, participants with CES-D scores ≥16 were classified as probable depression [43]. The cutoff point is significantly related to clinical assessments of depression [42, 44], and can predict depression diagnosis [45, 46]. The Cronbach’s alpha in the current sample was .90 at T1, .92 at T2, .89 at T3, and .78 at T4.

#### Background information

The information of sex, residence, one-child family, and major were recorded at baseline. Whether the participants had been quarantined and their residence had been under lockdown during COVID-19 were also recorded at T4.

#### Data analyses

The levels of the two outcomes, SPA and depressive symptoms at T1, T2, T3 and T4, were compared by one-way repeated measures ANOVA and post hoc tests. The associations between background variables and outcomes at T4 were tested by t-test or ANOVA, as appropriate. Spearman/Pearson’s correlation analyses were conducted to show the associations among COVID-related factors (i.e., quarantine, lockdown, boredom, emotional loneliness, and social loneliness during COVID-19) and SPA/depressive symptoms at T4. Simple linear regression was performed to identify the significant COVID-related factors of SPA at T4. In addition, the relationships between the COVID-related factors and SPA at T4 when the significant background variables of SPA and the level of SPA at T3 were adjusted for were also reported. Finally, multiple regression (hierarchical) was performed when all the COVID-related factors were included and the significant background variables of SPA, depressive symptoms, and the level of SPA at T3 were adjusted for. It is to identify the conjunction effects of all the potential COVID-related factors on SPA. The same regression analyses were also applied when the outcome was depressive symptoms. Observed power for the multiple regression analysis was over 90% with SPA and depressive symptoms as the outcome, respectively. The assumptions of these tests (e.g., continuous dependent variable, normal distribution of dependent variable) were checked and met. All the analyses were performed using SPSS 21.0. The level of statistical significance was .05.

## Results

### Changes in SPA and depressive symptoms over time

**Fig. 1:**
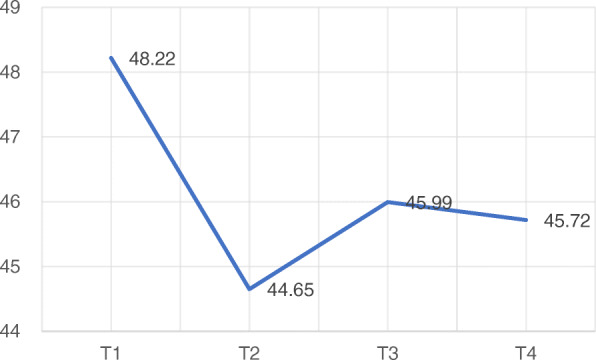
Levels of smart phone addiction at T1, T2, T3 and T4. Note: Level data are the mean scores of smart phone addiction among the 195 college students; Post hoc tests showed that the level of smart phone addiction at T1 (48.22) was significantly higher than that at T2, T3, or T4 (44.65, 45.99, 45.72) (*p* < .05). The levels of SPA at T2, T3 and T4 were not significantly different (*p* > .05)

**Fig. 2:**
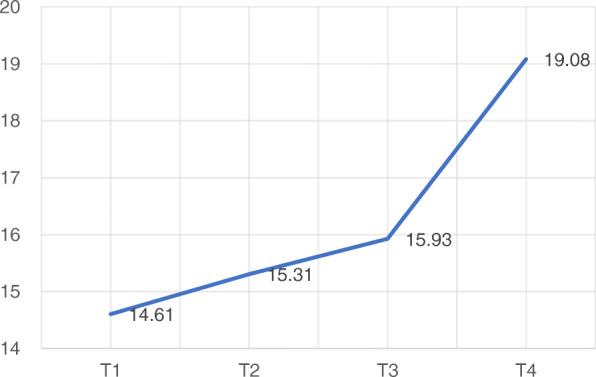
Levels of depressive symptoms at T1, T2, T3 and T4. Note: Level data are the mean scores of depressive symptoms among the 195 college students; Post hoc tests showed that the level of depressive symptoms at T4 (19.08) was significantly higher than that at T1, T2, or T3 (14.61, 15.31, 15.93) (*p* < .001). The levels of depressive symptoms at T1, T2, and T3 were not significantly different (*p* > .05)

**Fig. 3:**
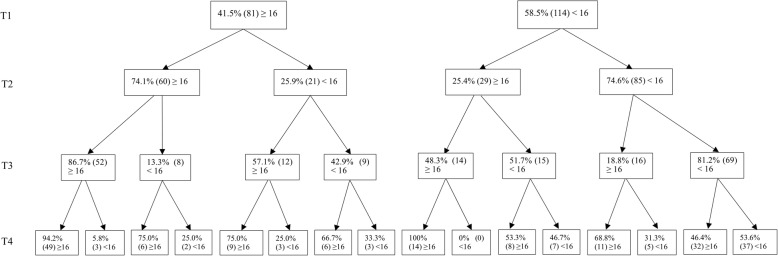
Prevalence and conversion rate of probable depression (CES-D ≥ 16) at T1, T2, T3 and T4

Table 2, Figs. 1 and 2 present the levels of SPA and depressive symptoms at four time points. The level of SPA at T1 (48.22) was significantly higher than that at T2, T3, or T4 (44.65, 45.99, 45.72) (*p* < .05). The levels of SPA at T2, T3 and T4 were not significantly different (*p* > .05). The level of depressive symptoms at T4 (19.08) was significantly higher than that at T1, T2, or T3 (14.61, 15.31, 15.93) (*p* < .001). The levels of depressive symptoms at T1, T2, and T3 were not significantly different (*p* > .05). Prevalence of probable depression was 41.5% at T1, 45.6% at T2, 48.2% at T3, and 69.2% at T4 (Fig. 3). As many as 49 students (25.1%) experienced probable depression at all four time points. Only 37 (19.0%) were not classified as having probable depression at all four time points. The incidence rates were 25.4% from T1 to T2, 26.4% from T2 to T3, and 51.5% from T3 to T4. The remission (defined as having CESD scores changed from ≥16 at baseline to < 16 at follow-up, 44) rates were 25.9% from T1 to T2, 25.8% from T2 to T3, and 11.5% from T3 to T4. SPA and depressive symptoms were positively correlated with each other at each time point.

**Table 2:** Levels of smart phone addiction and depressive symptoms at T1, T2, T3 and T4

	T1	T2	T3	T4	Group comparison
SPA					*F* = 8.29^***^ (T1 > T2, T3, T4)
Mean	48.22	44.65	45.99	45.72	
SD	10.60	11.31	12.71	11.63	
DS					*F* = 22.49^***^ (T1, T2, T3 < T4)
Mean	14.61	15.31	15.93	19.08	
SD	8.35	9.30	9.97	6.63	
r between SPA and DS	.39^***^	.45^***^	.51^***^	.44^***^	

One-way repeated measures ANOVA analyses; Pearson’s correlation analyses; *SPA* Smart phone addiction, *DS* Depressive symptoms; ^***^p < .001

### Associations between background/COVID-related variables and SPA/depressive symptoms at T4

Gender was significantly associated with depressive symptoms (Mean = 17.26 in males and Mean = 20.38 in females, t = 3.33, *p* < .001).

Table 3 presents the Spearman/Pearson’s correlations among COVID-related variables, SPA and depressive symptoms. Boredom (*r* = .38, *p* < .001) and emotional loneliness (*r* = .32, *p* < .001) during COVID-19 were significantly and positively correlated with SPA. Boredom (*r* = .31, *p* < .001), emotional loneliness (*r* = .29, *p* < .001), and social loneliness (*r* = .20, *p* < .01) during COVID-19 were significantly and positively correlated with depressive symptoms.

**Table 3:** Correlations among COVID-related factors, smart phone addiction and depressive symptoms at T4

	1	2	3	4	5	6	7
1 Quarantine	1						
2 Lockdown	.01	1					
3 Boredom	.11	.15^*^	1				
4 Emotional loneliness	−.03	.01	.39^***^	1			
5 Social loneliness	−.03	.05	−.00	.13	1		
6 SPA	.11	.10	.38^***^	.32^***^	.01	1	
7 DS	.01	.09	.31^***^	.29^***^	.20^**^	.44^***^	1

Spearman/Pearson’s correlation analyses; Quarantine and lockdown were dummy coded in the analyses; *SPA* Smart phone addiction, *DS* Depressive symptoms; ^*^p < .05; ^**^p < .01; ^***^p < .001

Tables 4 and 5 show the associations between COVID-related variables and SPA/depressive symptoms. Boredom and emotional loneliness during COVID-19 were significantly associated with SPA in all the regression models (*p* < .05). Quarantine, social loneliness and lockdown were not significantly associated with SPA (*p* > .05). All the factors explained 58% of the variance in SPA. Similar results were found when controlling for depressive symptoms. In addition, boredom, emotional loneliness, and social loneliness during COVID-19 were significantly associated with depressive symptoms in all the regression models (*p* < .05). Quarantine and lockdown were not significant factors of depressive symptoms (*p* > .05). All the factors explained 40% of the variance in depressive symptoms. Similar results were found when controlling for SPA.

**Table 4:** Linear regression model on smart phone addiction at T4

Variables	Block 1		Block 2		Block 3		Block 4	
	β	t	β	t	β	t	β	t
Quarantine	.10	1.37	.10	1.86	.08	1.74	.09	1.84
Lockdown	.11	1.52	.06	1.22	.03	.66	.03	.57
Boredom	.38	5.62^***^	.26	5.25^***^	.19	3.59^***^	.15	2.93^**^
Emotional loneliness	.32	4.63^***^	.22	4.38^***^	.15	2.79^**^	.12	2.28^*^
Social loneliness	.01	.17	.02	.45	.01	.13	−.03	−.58
*R*^2^					.58		.60	
*Df*					6/193		7/193	
*F*					42.18^***^		39.90^***^	

*SPA* Smart phone addiction; Block 1: simple regression analyses; Block 2: simple regression analyses adjusting for SPA at T3; Block 3: multiple regression analysis with multiple independent variables (all the COVID-related factors and the level of SPA at T3) being included; Block 4: multiple regression analysis with multiple independent variables (all the COVID-related factors, the level of SPA at T3 and depressive symptoms at T4) being included; ^*^p < .05; ^**^p < .01; ^***^p < .001

**Table 5:** Linear regression model on depressive symptoms at T4

Variables	Block 1		Block 2		Block 3		Block 4	
	β	t	β	t	β	t	β	t
Quarantine	−.01	−.14	.00	.00	.00	−.04	−.02	−.32
Lockdown	.09	1.27	.00	−.01	−.04	−.71	−.05	−.78
Boredom	.31	4.55^***^	.24	4.13^***^	.19	2.95^**^	.14	2.20^*^
Emotional loneliness	.29	4.28^***^	.25	4.18^***^	.17	2.65^**^	.13	2.09^*^
Social loneliness	.20	2.87^**^	.15	2.43^*^	.14	2.42^*^	.15	2.64^**^
*R*^2^					.40		.42	
*Df*					7/193		8/193	
*F*					17.68^***^		16.98^***^	

*SPA* Smart phone addiction; Block 1: simple analyses; Block 2: simple analyses adjusting for gender and depressive symptoms at T3; Block 3: multiple regression analysis with multiple independent variables (all the COVID-related factors and the level of depressive symptoms at T3) being included; Block 4: multiple regression analysis with multiple independent variables (all the COVID-related factors, the level of depressive symptoms at T3 and SPA at T4) being included; ^*^p < .05; ^**^p < .01; ^***^p < .001

## Discussion

To our knowledge, this is the first longitudinal study to track the changes in SPA and depressive symptoms before and during COVID-19. It is interesting to find different patterns in the changes of SPA and depressive symptoms over time.

### Changes in SPA and depressive symptoms over time

First, the level of SPA was greatest at T1 (the first semester of Year 1) and significantly decreased since T2 (the second semester of Year 1). It may be because freshmen in the first semester of Year 1 may need to deal with various new challenges and stress, including college adjustment and cultural adjustment [47], and had few coping resources in this new environment; smart phone use may become a major and handy means of coping with their stress [48]. As they gradually adjust into college life and gain more coping resources during the follow-up survey period, the dependence of smart phone may decrease and maintain at a stable level. Future studies may examine whether increased college adjustment and coping resource gain would be mechanisms/mediators of the change in SPA levels over time. However, we did not find any significant differences in the levels of SPA between T2/T3 and T4. The results may suggest that COVID-19 did not have significant impact on SPA. It does not seem to support the consensus highlighted by recent commentaries and position papers [3, 19–21]. One plausible explanation may be that the measure of SPA stresses the withdrawal symptoms and negative consequences of smart phone use (e.g., causing troubles and decreasing efficiency due to smart phone use) [49], and the participants may be less likely to recognize or endorse such symptoms which is due to the fact that over-engagement with smart phone are seen normal during the COVID-19 isolation. It is anticipated that the perceived negative consequences of SPA would increase when COVID-19 has passed and people have experienced difficulties to re-adaptation. Further follow-up studies are warranted to monitor the changes in SPA and other technology-related disorders and behavioral addiction (e.g., social media addiction, internet gaming disorder) when the COVID-19 pandemic has passed.

Second, depressive symptoms and prevalence of probable depression did not vary at T1, T2, and T3 but substantially increased at T4. People were more likely to become probably depressed during COVID-19 than pre-COVID (Incidence rate: 51.5% versus 25.4–26.4%. People with probable depression before COVID-19 were also less likely to remit during COVID-19 (Remission rate: 11.5% versus 25.9–25.8%). These results provide robust evidence to support the argument that probable depression has substantially increased during COVID-19 compared to pre-COVID. This finding is consistent with the assertion in a number of recent commentary papers and cross-sectional studies that the COVID-19 outbreak has threatened mental health of people globally (e.g., [3, 8–11, 50, 51]). Follow-up studies should track whether these cases of probable depression would remit or not with the progression of the COVID-19 epidemic and estimate the needs of mental health care in the study population. It is also worth noting that the study population also showed high prevalence of probable depression (41.5–48.2%) before COVID-19. Also, 25.1% experienced probable depression at all four time points, but only 19.0% did not. It suggests that this population may be a high risk group of probable depression. Indeed, college life is a period filled with new changes and instability (e.g., college maladjustment, exam stress, interpersonal problems) [47] which may significantly contribute to the high level of probable depression. It may be also because the participants were medical students; studies from other parts of world have also shown a high prevalence of depression in population (e.g., [52, 53]). Although probable depression may not necessarily convert to clinical depression, such a high proportion of students with probable depression still needs to draw attention from health workers and educators.

### Associations between COVID-related variables and SPA/depressive symptoms

The negative emotions experienced during COVID-19 may explain depressive symptoms and SPA at T4. State boredom and loneliness are especially relevant during an epidemic outbreak, where lockdown, quarantine, and social distancing reduce the range of possible activities, such as traveling, physical activities, and face-to-face interpersonal interactions [54]. Our study suggested that greater state boredom, emotional loneliness, and social loneliness were associated with higher level of depressive symptoms during the outbreak, which is consistent with previous study suggesting boredom proneness and loneliness to be significant predictors of depression [55, 56]. This study extends the previous research by revealing the negative impact of state boredom and loneliness even in the disaster context such as the COVID-19 pandemic which may put individuals’ life at risk. Furthermore, we found that boredom and emotional loneliness during COVID-19 were also associated with SPA at T4. The results may support the application of the general strain theory [29], which suggests that external stress and negative emotions can lead to behavioral problems, to explain the development of SPA during COVID-19. That is, SPA could be a consequence of relying on smart phone to relive or escape from emotional loneliness and boredom induced by the pandemic. Future work should track the changes in these emotions and test whether such emotions would have long-term effects on individuals’ well-being after the pandemic.

Such emotional variables are modifiable and thus can be used to guide interventions to prevent and reduce mental and behavioral problems, such as depressive symptoms and SPA. Mental health professionals should be aware of how these psychological vulnerabilities during the pandemic may contribute to these problems, provide emotional support, and advice alternative social/recreational activities, all of which could be essential to minimizing the risks of depression and SPA.

### Limitations and future studies

The study has several limitations. First, the study used a convenience sample of college students and thus the generalization of these findings into other populations should be cautious. Future research may explore the proposed model among diverse populations. The survey had a relatively small sample size. Future studies should also enlarge the sample size. Secondly, this study relied on self-report measures. Future studies could employ multiple informants (e.g., peers and parents), objective stress measurements (e.g. blood pressure during smart phone use) or brain imaging approaches (e.g. brain activity patterns during smart phone use) to collect data in order to validate the findings. It is also a limitation that this study used the CES-D scores 16 as thresholds of the outcome measures. CES-D cannot be used as an isolated diagnostic assessment of depression, although its 16 cut-off score has acceptable screening accuracy for probable depression in the general population [57]. Diagnostic measures and clinical sample should be used in future work to validate the findings. Thirdly, although the study was particularly interested in the emotional status and their relationships with mental and behavioral problems during COVID-19, it is equally important to test them prior to the pandemic which is a limitation of this study. Fourthly, only one-wave investigation was conducted during COVID-19. Mental and behavioral status could vary significantly with the progression of the epidemic and should be followed up at different stages of the epidemic. Last but not least, this study did not investigate the purposes of smartphone use. Smartphone use for different purposes (e.g., educational purposes vs. social media purposes) may have different effects on users’ mental health and may play as a moderator. Future work should take this variable into consideration.

## Conclusion

In sum, we found a substantial increase in depressive symptoms but an insignificant change in SPA before and during COVID-19. Furthermore, boredom and loneliness were significantly associated with depressive symptoms and SPA during COVID-19. The identified emotional factors could be used to reduce depressive symptoms during COVID-19 and prevent the potential risk of SPA.

## Supplementary Information

**Additional file 1.** English version of the questionnaire.

## Data Availability

The datasets used and/or analyzed during the current study are available from the corresponding author on reasonable request.

## Abbreviations

COVID-19: Coronavirus disease 2019
SPA: Smart phone addiction
CES-D: Center for Epidemiologic Studies Depression Scale
