# Supplementary material for: A longitudinal study of changes in smart phone addiction and depressive symptoms and potential risk factors among Chinese college students
Source: BMC Psychiatry. 2021 May 14;21:252. doi: 10.1186/s12888-021-03265-4 (PMC8120756; doi:10.1186/s12888-021-03265-4)
Supplement: Supplementary file 1 — Additional file 1. English version of the questionnaire. [file 12888_2021_3265_MOESM1_ESM.docx]

English version of the questionnaire

| **During COVID-19, you felt…** | Strongly disagree | Disagree | Neutral | Agree | Strongly agree |
| --- | --- | --- | --- | --- | --- |
| a. I felt bored. | 1 | 2 | 3 | 4 | 5 |
| b. I was easily distracted. | 1 | 2 | 3 | 4 | 5 |
| c. Time was passing by slower than usual. | 1 | 2 | 3 | 4 | 5 |

| **During COVID-19, you felt…** |  |  |  |
| --- | --- | --- | --- |
| a. I experience a general sense of emptiness | **Yes** | **More or less** | **No** |
| b. I miss having people around | **Yes** | **More or less** | **No** |
| c. I often feel rejected | **Yes** | **More or less** | **No** |
| d. There are plenty of people I can rely on when I have problems | **Yes** | **More or less** | **No** |
| e. There are many people I can trust completely | **Yes** | **More or less** | **No** |
| f. There are enough people I feel close to | **Yes** | **More or less** | **No** |

**The Center for Epidemiological Studies-Depression**

| **In the latest week** | **a little,**  **（less than 1 day）** | **some**  **（1—2 days）** | **a good**  **part of the time（3—4 days）** | **most of the time（5—7 days）** |
| --- | --- | --- | --- | --- |
| 1. I was bothered by things that usually don't bother me | 0 | 1 | 2 | 3 |
| 2. I did not feel like eating; my appetite was poor | 0 | 1 | 2 | 3 |
| 3. I felt that I could not shake off the blues even with help from my family or friends | 0 | 1 | 2 | 3 |
| 4. I felt that I was just as good as other people | 0 | 1 | 2 | 3 |
| 5. I had trouble keeping my mind on what I was doing | 0 | 1 | 2 | 3 |
| 6. I felt depressed | 0 | 1 | 2 | 3 |
| 7. I felt that everything I did was an effort | 0 | 1 | 2 | 3 |
| 8. I felt hopeful about the future | 0 | 1 | 2 | 3 |
| 9. I thought my life had been a failure | 0 | 1 | 2 | 3 |
| 10. I felt fearful | 0 | 1 | 2 | 3 |
| 11. My sleep was restless | 0 | 1 | 2 | 3 |
| 12. I was happy | 0 | 1 | 2 | 3 |
| 13. I talked less than usual | 0 | 1 | 2 | 3 |
| 14. I felt lonely | 0 | 1 | 2 | 3 |
| 15. People were unfriendly | 0 | 1 | 2 | 3 |
| 16. I enjoyed life | 0 | 1 | 2 | 3 |
| 17. I had crying spells | 0 | 1 | 2 | 3 |
| 18. I felt sad | 0 | 1 | 2 | 3 |
| 19. I felt that people disliked me | 0 | 1 | 2 | 3 |
| 20. I could not get "going" | 0 | 1 | 2 | 3 |

**The mobile phone addiction index (MPAI) scale**

**Instruction: Each of the following questions has 5 choices. 1-5 indicates the degree from low to high. There is no right or wrong answer. Please tick the number (option) at the end of each question that corresponds to your actual situation.**

1. Your friends and family complained about your use of the mobile phone 1 2 3 4 5

2. You have been told that you spend too much time on your mobile phone 1 2 3 4 5

3. You have tried to hide from others how much time you spend on your mobile phone 1 2 3 4 5

4. You have received mobile phone bills you could not afford to pay 1 2 3 4 5

5. You find yourself engaged on the mobile phone for longer period of time than intended 1 2 3 4 5

6. You have attempted to spend less time on your mobile phone but are unable to 1 2 3 4 5

7. You can never spend enough time on your mobile phone 1 2 3 4 5

8. When out of range for some time, you become preoccupied with the thought of missing a call 1 2 3 4 5

9. You find it difficult to switch off your mobile phone 1 2 3 4 5

10. You feel anxious if you have not checked for messages or switched on your mobile phone for some time 1 2 3 4 5

11. You feel lost without your mobile phone 1 2 3 4 5

12. If you don't have a mobile phone, your friends would find it hard to get in touch with you 1 2 3 4 5

13. You have used your mobile phone to talk to others when you were feeling isolated 1 2 3 4 5

14. You have used your mobile phone to talk to others when you were feeling lonely 1 2 3 4 5

15. You have used your mobile phone to make yourself feel better when you were feeling down 1 2 3 4 5

16. You find yourself occupied on your mobile phone when you should be doing other things, and it causes problem 1 2 3 4 5

17. Your productivity has decreased as a direct result of the time you spend on the mobile phone 1 2 3 4 5
